# Supplementary material for: Spatiotemporal persistence of multiple, diverse clades and toxins of Corynebacterium diphtheriae
Source: Nat Commun. 2021 Mar 8;12:1500. doi: 10.1038/s41467-021-21870-5 (PMC7940655; doi:10.1038/s41467-021-21870-5)
Supplement: Supplementary file 1 — Supplementary Information [file 41467_2021_21870_MOESM1_ESM.pdf]

Supplementary Table 1: Primer used for in-silico PCR of the *tox* gene in *Corynebacterium diphtheriae* genomes.

	Forward	Reverse
**tox**	GTGAGCAGAAAACTGTTTGCGTCAA	TCAGCTTTTGATTTCAAAAAATAGC

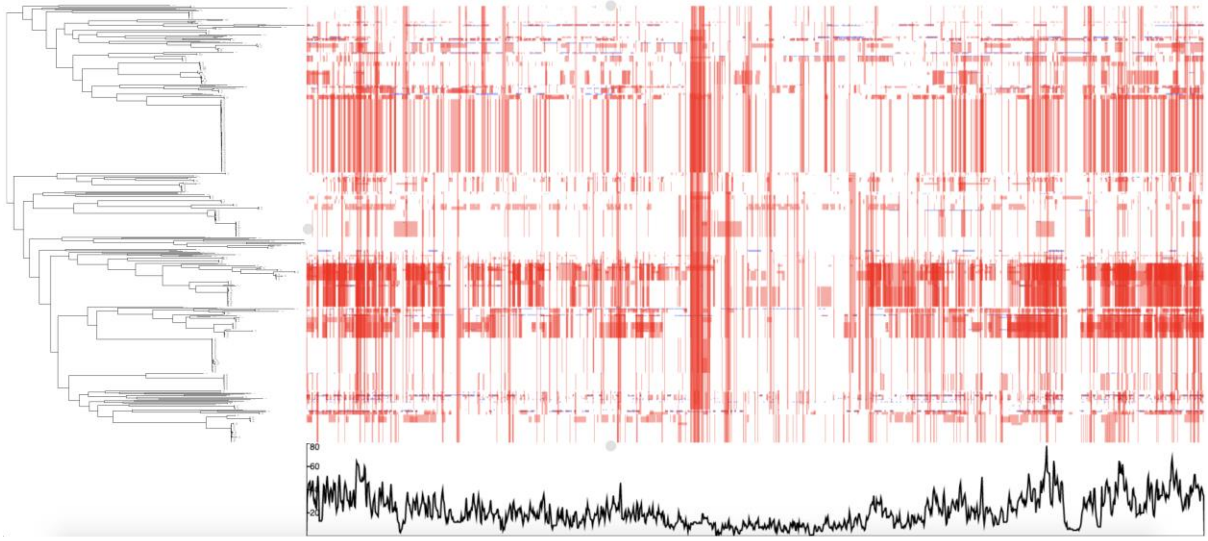

Supplementary Figure 1: Representation of recombination in the core genes of global *Corynebacterium diphtheriae* isolates positioned in the phylogeny. Red colour blocks indicate ancestral events, while blue blocks indicate events that only affect a single isolate.

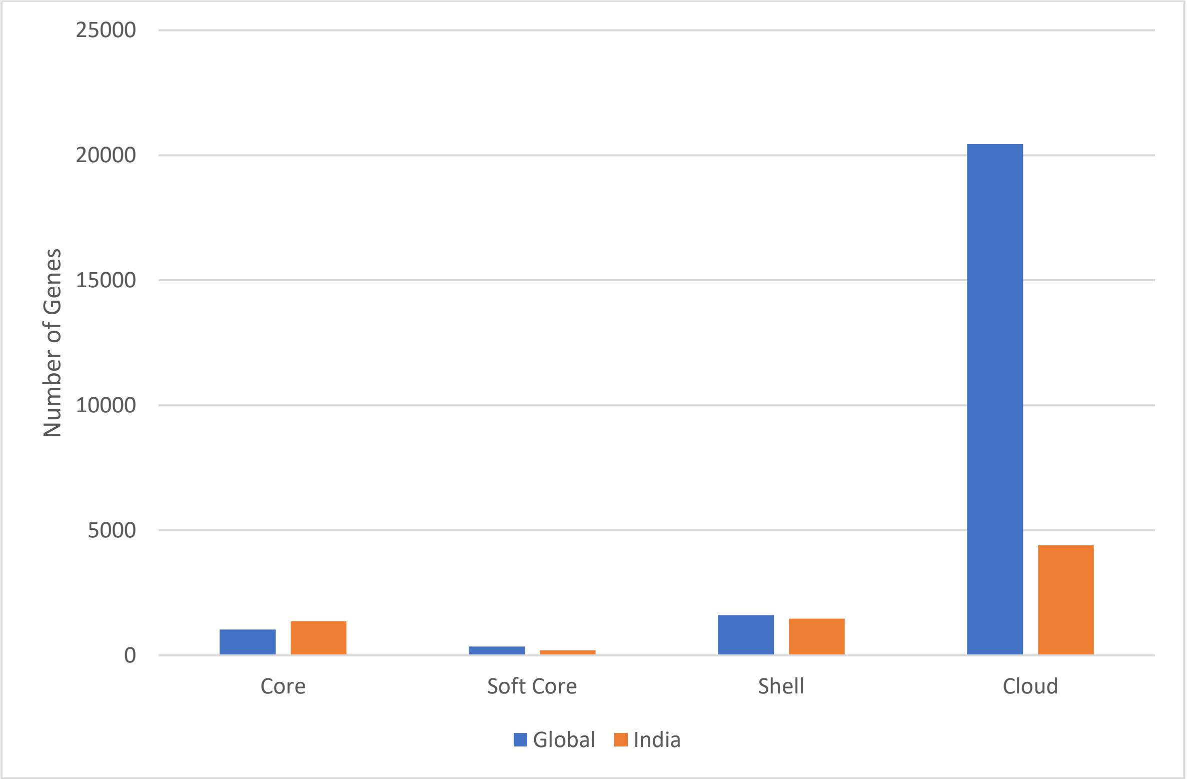

Supplementary Figure 2: Genes identified by Roary and Roary Plots, with the global collection in blue and the Indian subset in orange. Source data are provided as a Source Data file.

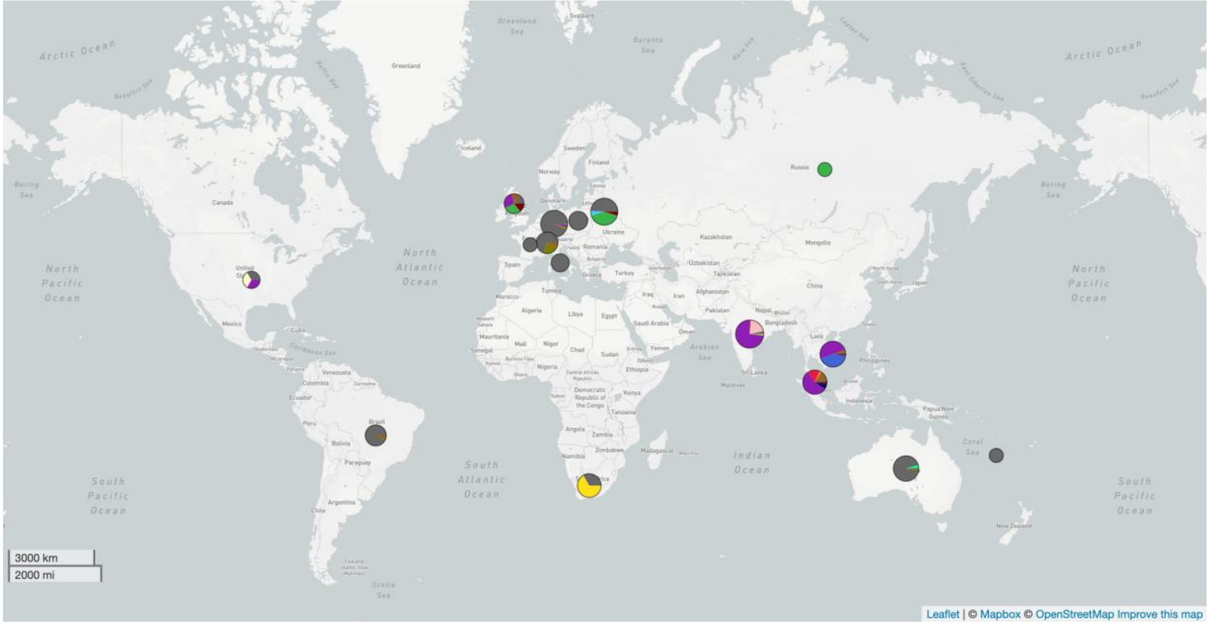

Supplementary Figure 3: *tox* gene group variants shown by country of isolation.

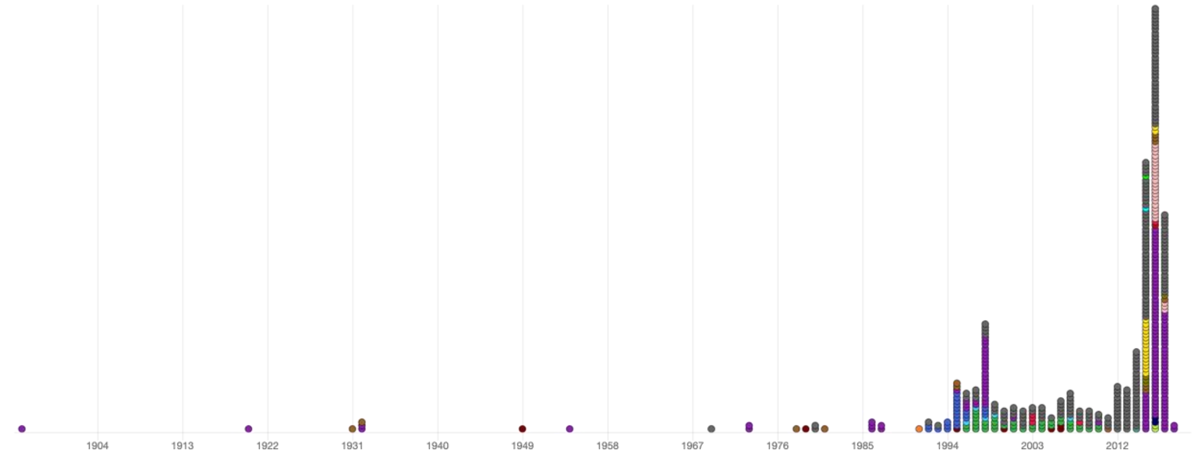

Supplementary Figure 4: *tox* gene group variants shown by year of isolation.

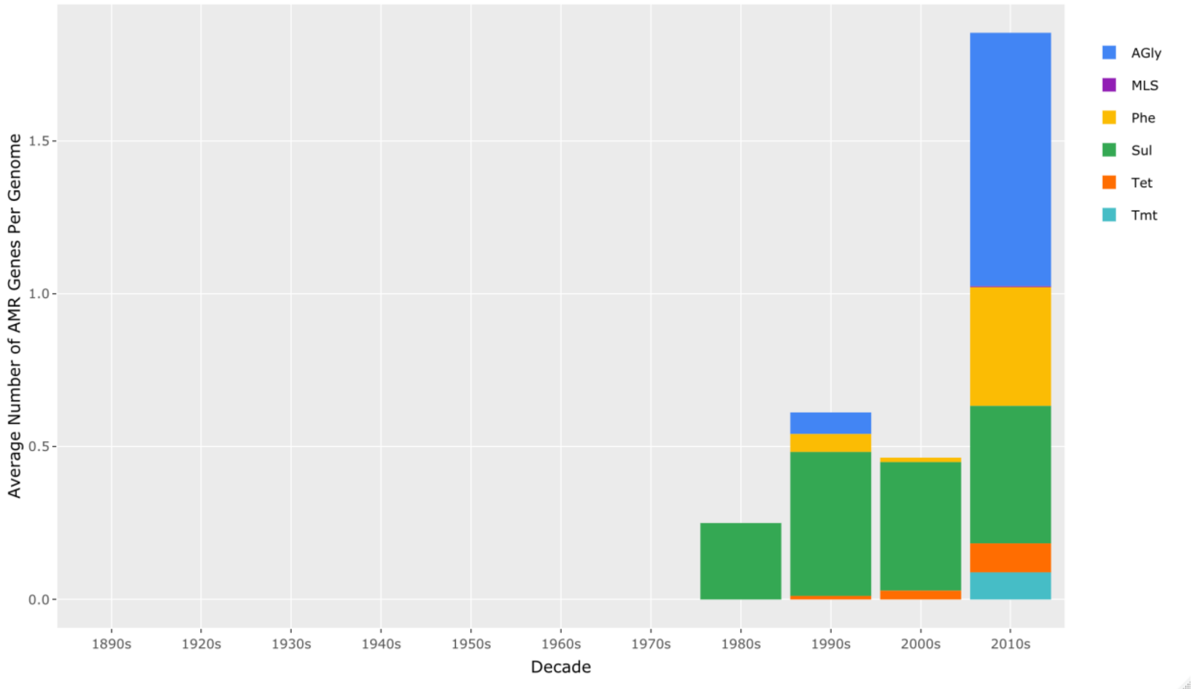

Supplementary Figure 5: Antimicrobial resistance by decade. The coloured bars represent the average number of genes per genome found that resist each class of antibiotic per decade. AGly (blue) = aminoglycosides, MLS (purple) = macrolide-lincosamide-streptogramin, Phe (yellow) = phenicols, Sul (green) = sulfonamides, Tet (orange) = tetracyclines, Tmt (light blue) = trimethoprim. Source data are provided as a Source Data file.

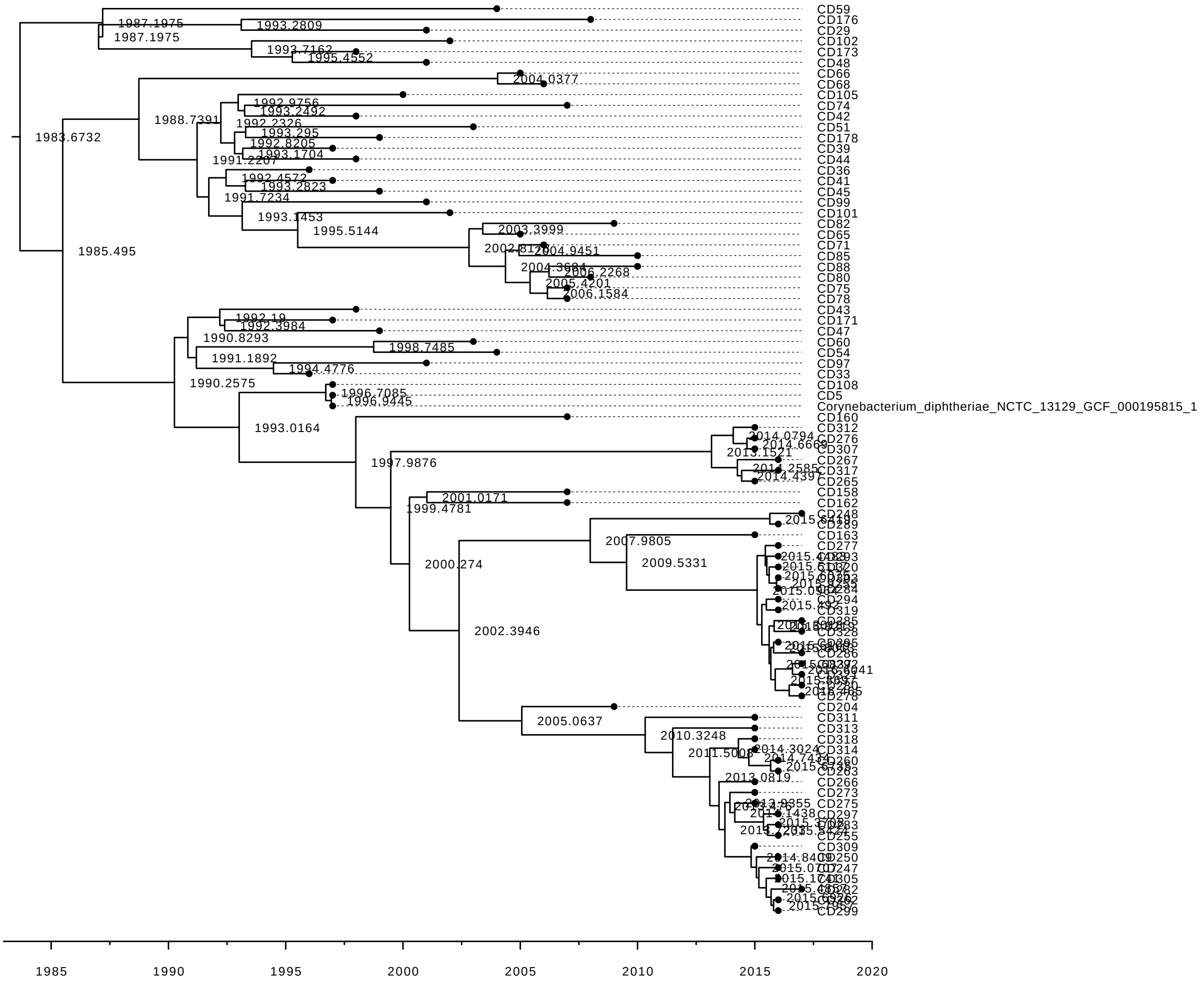

Supplementary Figure 6: BEAST phylogeny of the monophyletic European group.

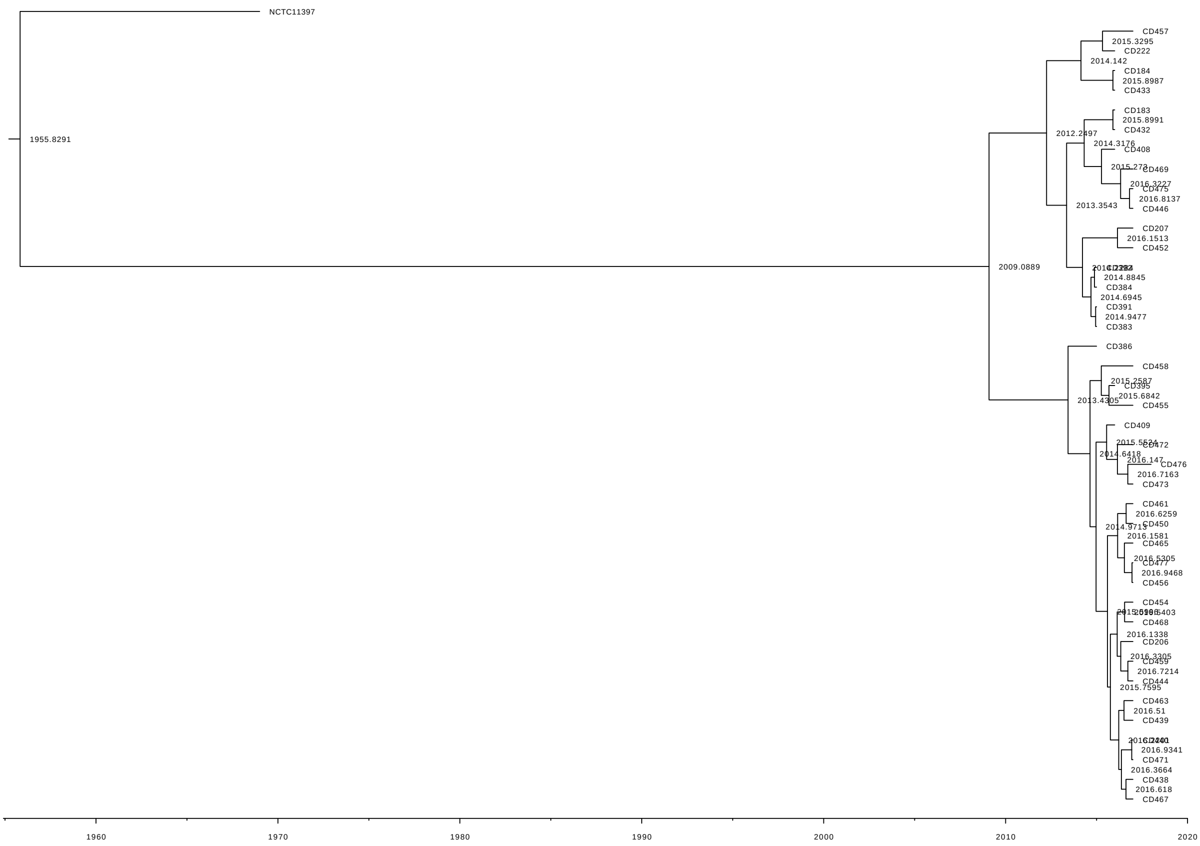

Supplementary Figure 7: BEAST phylogeny of the monophyletic Indian group.

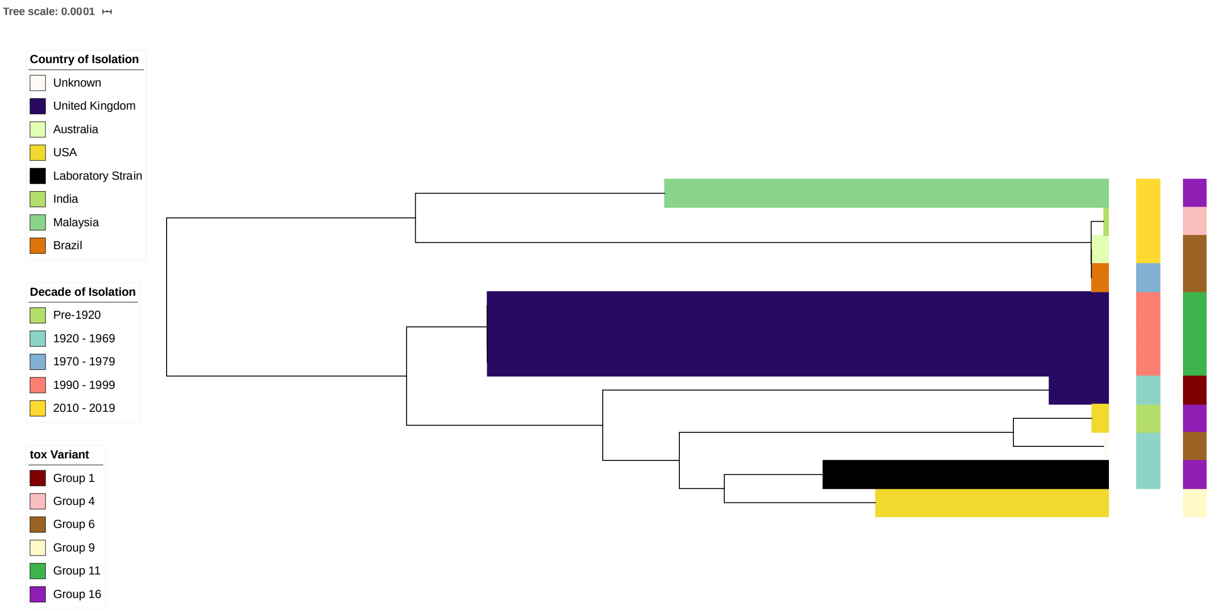

Supplementary Figure 8: Maximum likelihood phylogeny of corynephage from 11 toxigenic completed genomes, showing the country and decade of isolation along with the *tox* gene variant carried.
