# Supplementary material for: Spatiotemporal persistence of multiple, diverse clades and toxins of Corynebacterium diphtheriae
Source: Nat Commun. 2021 Mar 8;12:1500. doi: 10.1038/s41467-021-21870-5 (PMC7940655; doi:10.1038/s41467-021-21870-5)
Supplement: Supplementary file 3 — Descriptions of Additional Supplementary Files [file 41467_2021_21870_MOESM3_ESM.pdf]

Descriptions of Additional Supplementary Files

Supplementary Data 1

Description: Information on all isolates included in this study. Assigned code, name, accession, year and location of isolation is included, along with MLST designation determined using MLSTcheck, tox group variants present were determined using in-silico PCR and the antimicrobial resistance genes present determined using ARIBA. Phenotypic antimicrobial resistance testing was carried out on the 61 novel Indian isolates (Ampicillin (AM), Azithromycin (AZ), Ciprofloxacin (CI), Chloramphenicol (CL), Clindamycin (CM), Cefotaxime (CT), Erythromycin (EM), Gentamycin (GM), Imipenem (IP), Linezolid (LZ), Moxyfloxacin (MX), Penicillin (PG), Rifampicin (RI), Tetracycline (TC), Ceftriaxone (TX), Vancomycin (VA), Trimethoprime-Sulphamethoxazole (SXT), Amoxicillin/clavulanic acid (XL), Doxycycline (DC).
